# Supplementary material for: Aqueous Extract of Freshwater Clam Increases Alcohol Metabolism in Rats in a Preclinical Model
Source: Nutrients. 2025 Jun 3;17(11):1915. doi: 10.3390/nu17111915 (PMC12157754; doi:10.3390/nu17111915)
Supplement: Supplementary file 1 [file nutrients-17-01915-s001.zip › nutrients-3596681-supplementary.pdf]

Supplementary material

1. Calculation formula of ethanol concentration:

$$[\text{Ethanol}] = \frac{OD_{SAMPLE} - OD_{BLANK}}{\text{Slope}} \times n\ (\%)$$

where $\mathbf{OD_{SAMPLE}}$ and $\mathbf{OD_{BLANK}}$ are the OD_{565}nm values of the sample and blank. n is the dilution factor.

2. Calculation formula of ALDH activity:

Calculate the OD increase by the test samples: $\Delta OD = A_1 - A_0$

Apply the ΔOD to the NADH standard curve to get B nmol of NADH generated by ADH during the reaction time ($\Delta T = T_2 - T_1$).

$$\text{ADH Activity} = \frac{B}{\Delta T \times V} \times \text{Sample Dilution Factor} = \text{nmol/min/ml} = \text{mU/mL}$$

Where:

B is the NADH amount generated by ADH (in nmol).

T is the time of reaction (in minute).

V is the sample volume added into the reaction well (in mL).

3. Calculation formula of ALDH activity:

Apply sample ΔOD 450nm [(A2 - A2B) - (A1 - A1B)] to the standard curve to get B nmol of NADH generated during the reaction time (ΔT = T2 - T1).

$$\text{ALDH Activity} = \frac{B}{(\Delta T \times V)} \times \text{Dilution Factor} = \text{nmol/min/ml/} = \text{mU/ml}$$

Where:

B is the amount of NADH generated by your sample (nmol).

ΔT is the reaction time (min).

V is the sample volume used in the reaction well (mL).

4. Analysis procedure of CAT activity:
   (1) Prepare formaldehyde standards to create a formaldehyde standard curve.
   (2) Add 100 μL of Assay Buffer (100 mM potassium phosphate, pH =7.0), 30 μL of methanol, and 20 μL of formaldehyde standard or sample into the designated wells.
   (3) Initiate the reaction by adding 20 μL of 35 mM hydrogen peroxide to all wells.
   (4) Cover the plate with the plate cover and incubate on a shaker for 20 min at room temperature.
   (5) Add 30 μL of potassium hydroxide to each well to terminate the reaction and then add 30 μL of Catalase Purpald (Chromogen) to each well.
   (6) Cover the plate and incubate for 10 min at room temperature on the shaker.
   (7) Add 10 μL of Catalase Potassium Periodate to each well. Cover with plate cover and incubate five min at room temperature on a shaker.
   (8) Read the absorbance at 540 nm using a plate reader. The formaldehyde concentration of the samples was then calculated based on the standard curve.
   (9) Calculate the CAT activity of the sample using the following equation.

$$\text{CAT Activity} = \frac{\mu\text{M of Sample}}{20\text{ min.}} \times \text{Sample dilution} = \text{nmol/min/ml}$$

5. Analysis procedure of SOD activity:
   (1) Prepare SOD standards to create a formaldehyde standard curve.
   (2) Add 200 μL of diluted Radical Detector and 10 μL of standard or sample into the designated wells.
   (3) Initiate the reaction by adding 20 μL of diluted Xanthine Oxidase to all wells.
   (4) Carefully shake the plate for a few seconds to mix. Cover with the plate cover.
   (5) Incubate the plate on a shaker for 30 minutes at room temperature. Read the absorbance at 440-460 nm using a plate reader.
   (6) Plot the linearized SOD standard rate (LR) as a function of final SOD Activity (U/ml).
   (7) Calculate the SOD activity of the sample using the following equation.

$$\text{SOD (U/ml)} = \left[\left(\frac{\text{sample LR - y-intercept}}{\text{slope}}\right) \times \frac{0.23\text{ ml}}{0.01\text{ ml}}\right] \times \text{sample dilution}$$
